# Temperature Management Guidelines

**Published:** 2015

**Authors:** John W. Hammon, Linda Shore-Lesserson, Timothy A. Dickinson

**Affiliations:** * Department of Cardiothoracic Surgery, Wake Forest University School of Medicine, Winston-Salem, North Carolina; † Department of Anesthesiology, Hofstra Northshore-Long Island Jewish Medical School, New Hyde Park, New York; ‡ Clinical Performance Improvement, SpecialtyCare, Nashville, Tennessee

The guideline in this issue, “The Society of Thoracic Surgeons, The Society of Cardiovascular Anesthesiologists, and The American Society of ExtraCorporeal Technology: Clinical practice guidelines for cardiopulmonary bypass—Temperature management during adult cardiopulmonary bypass” represents the first multidisciplinary effort to develop guidelines for the practice of cardiopulmonary bypass (CPB) (1). In the light of the rapid advances in technology and increased complexity of cardiac operations that use CPB, the development of guidelines is necessary to establish a foundation for the evidence base.

When this process began, individuals were selected by the Society of Thoracic Surgeons, the Society of Cardiovascular Anesthesiologists, and the American Society of ExtraCorporeal Technology to form a multidisciplinary writing group and construct a series of guidelines. The group agreed to review the appropriate literature relative to CPB from 2001 through 2014. John W. Hammon was selected to represent the Society of Thoracic Surgeons, Linda Shore-Lesserson was selected to represent the Society of Cardiovascular Anesthesiologists, and Timothy A. Dickinson was selected to represent the American Society of ExtraCorporeal Technology. We have expanded and formed a large team of experts in this field to review the relevant literature, assess the levels of evidence, and synthesize the recommendations into appropriate and actionable clinical practice guidelines.

Robert A. Baker, PhD, and his group at Flinders University, Adelaide, South Australia, Australia, has developed a tool to facilitate the guideline development process. The tool, known as “Guideliner,” is an Internet-based program that stores literature searches and literature reviews, creates work lists, and directs the user through a very conservative evidence-review process. Thus far, the Guideliner has guided our multidisciplinary group through the review of 3,321 abstracts and 935 complete articles. Articles published from 2001 through 2014 have been carefully screened to find the most relevant and scientifically sound evidence. These would be combined with older high-quality publications to form future guidelines for CPB.

Dr. Richard Engelman and his team have produced a comprehensive statement regarding the best practices in CPB temperature management to date. Subsequently, we hope other guidelines will be presented, including renal protection, neurologic protection, anticoagulation management, equipment selection, and interdisciplinary collaboration; the final guideline would address the conduct of CPB and perfusion safety. Our expectation is that this series of guidelines will be used by clinical teams to facilitate the assessment and improvement of care for the patients whom we collectively serve.
